# Review of Cerebrospinal Fluid Isolates in Acute Bacterial Meningitis: Phenotypic Characterization and Antibiogram

**DOI:** 10.7759/cureus.108726

**Published:** 2026-05-12

**Authors:** Sanika S Chavan, Geeta S Karande, Satish R Patil

**Affiliations:** 1 Department of Microbiology, Krishna Vishwa Vidyapeeth (Deemed to be University), Karad, IND

**Keywords:** acute bacterial meningitis, bacterial meningitis, cerebrospinal fluid, haemophilus influenzae, neisseria meningitidis, severe sequelae, streptococcus pneumoniae

## Abstract

Acute bacterial meningitis (ABM) is a life-threatening infection that affects the brain and is associated with high morbidity and mortality. Prompt diagnosis and early initiation of appropriate antibiotics are crucial for favorable outcomes. Cerebrospinal fluid (CSF) analysis plays a crucial part in confirming the diagnosis and identifying the causative organisms. This review summarizes the phenotypic characterization of significant bacterial infections found in CSF, including *Streptococcus pneumoniae, Neisseria meningitidis, Haemophilus influenzae, *and *Listeria monocytogenes.* Additionally, current trends in antimicrobial susceptibility patterns and their clinical implications are discussed. Understanding these aspects is crucial for guiding empirical therapy, monitoring emerging resistance, and improving patient management strategies in cases of severe bacterial meningitis.

## Introduction and background

Acute bacterial meningitis (ABM) is a medical emergency, characterized by meningeal inflammation caused by bacterial invasion. Despite advances in antimicrobial therapy and vaccination programs, ABM continues to pose a significant global health burden. All age groups are vulnerable to the illness, which often leads to severe neurological complications or death if not treated promptly [1].

Fever, headache, stiff neck, and impaired mental status are typical clinical symptoms. Laboratory confirmation through cerebrospinal fluid (CSF) examination is essential for diagnosis, pathogen identification, and antimicrobial susceptibility testing (AST). The emergence of antibiotic-resistant strains has further complicated the management of ABM, making continuous monitoring of resistance patterns crucial [2].

The most common bacterial infections responsible for ABM are* Listeria monocytogenes, Neisseria meningitidis, Haemophilus influenzae, *and* Streptococcus pneumoniae.* Phenotypic characterization of these organisms involves microscopy, culture characteristics, and biochemical testing, which aid in accurate identification [2].

AST of CSF isolates is critical for detecting resistance patterns and guiding empirical and definitive therapy. Recent trends indicate increasing resistance to commonly used antibiotics, emphasizing the need for updated treatment guidelines. Preventive strategies such as conjugate vaccinations have considerably decreased the frequency of meningitis caused by certain pathogens, but emerging resistance remains a major concern [3,4].

Bacterial meningitis

Bacterial meningitis is a life-threatening medical emergency associated with high morbidity and mortality despite appropriate treatment [5]. It remains associated with high case fatality rates, causing an estimated 288,649 deaths globally each year, including approximately 94,833 deaths among children under five years. Bacterial meningitis generally causes major issues such as injury to the brain, hearing loss, and eventually death if not identified and addressed instantly. Up to 24% of the survivors experience long-term consequences, such as epilepsy, intellectual disability, or sensorineural deafness, particularly in situations where the disease develops in the early neonatal age [5].

During non-epidemic conditions, meningococcal infections are most common in children under school-age with 50-60% of cases being in children aged three months to five years [6]. The clinical signs and symptoms can't be always depended upon; laboratory support is imperative to achieve early diagnosis [7].

Meninges 

The meninges are three protective membranes that surround the central nervous system (CNS): the dura mater, arachnoid mater, and pia mater. The dura mater is the outermost, tough layer; the arachnoid mater is a thin, avascular membrane; and the pia mater is a delicate layer closely adherent to the brain and spinal cord. These layers form three spaces: the epidural, subdural, and subarachnoid spaces. The meninges function primarily to protect the brain and spinal cord and to support fluid circulation [8,9].

Cerebrospinal fluid 

Definition

CSF is a clear, colorless biological fluid produced mainly by the choroid plexus that circulates within the ventricular system and subarachnoid space, providing mechanical protection and metabolic support to the CNS [10].

Functions

CSF acts as a mechanical cushion and homeostatic regulator for the central nervous system. By providing buoyancy and cushioning, it reduces the brain’s effective weight from approximately 1500 g to 25-50 g [11]. It also helps regulate intracranial pressure (ICP) by compensating for fluctuations in blood volume within the skull during the cardiac cycle [12].

Additionally, it plays a vital role in maintaining CNS homeostasis by facilitating the transport of nutrients, hormones, and signaling molecules through the brain parenchyma. The choroid plexus epithelial cells are highly active and contain numerous organelles that enable the synthesis and secretion of proteins and peptides into this fluid [13]. It is also rich in hormones, neurotrophic factors, and growth factors that influence various CNS functions [13,14]. Furthermore, it acts as a “sink” by removing metabolic waste products from the brain along concentration gradients [14].

Another important but often overlooked function is immune surveillance. Although produced in the ventricles, it primarily circulates through the subarachnoid space (SAS), which accounts for approximately 75% of its flow within the CNS [15]. This space, located between the arachnoid and pia mater (leptomeninges), allows immune cells, predominantly T lymphocytes, to circulate and monitor for pathogens [16].

These immune cells enter through meningeal blood vessels and traverse the blood-meningeal barrier [17,18]. The relatively higher permeability of this barrier compared to the blood-brain barrier (BBB) facilitates immune cell migration due to the absence of astrocytic end-feet [19].

Types of meningitis

Acute Meningitis

Acute meningitis commonly presents with symptoms such as fever, neck stiffness, headache, nausea, vomiting, neurological abnormalities, and altered mental status. In severe bacterial meningitis, the cerebrospinal fluid typically shows a markedly elevated cell count (≈1000/mm³), predominantly polymorphonuclear leukocytes (PMNs). Compared to serum levels, it demonstrates reduced glucose concentration and increased protein levels. In healthy individuals, serum glucose ranges from 45 to 100 mg/dL, while its glucose concentration is usually about 60% of plasma glucose (≈45-80 mg/dL). Protein levels vary with age, ranging from approximately 15-50 mg/dL in adults and higher values in newborns.

Acute bacterial meningitis is a potentially life-threatening condition that requires prompt and specialized medical management. Although less common than viral meningitis, it is more severe and can be caused by a variety of pathogens. Prior to the 1990s, *Haemophilus influenzae* type b (Hib) was the leading cause; however, its incidence has significantly declined following the introduction of routine childhood vaccination. Currently, *N. meningitidis *and *S. pneumoniae* are the predominant causative organisms. A key shared virulence factor among these pathogens is the presence of a polysaccharide capsule, which enhances their ability to evade host immune responses [20,21]. Table 1 summarizes the CSF findings in different types of meningitis.

**Table 1:** Cerebrospinal fluid (CSF) findings in different types of meningitis Cerebrospinal fluid (CSF) results from bacterial and viral (aseptic) meningitis are compared in this table [20-22]. Increased protein levels, decreased glucose concentration, turbid or purulent fluid appearance, and a noticeably higher cell count with polymorphonuclear leukocyte (PMN) predominance are common signs of bacterial meningitis. Viral meningitis, on the other hand, is characterized by a slight to moderate increase in cell count with lymphocytic predominance, mildly raised protein, normal glucose levels, and a clear appearance of fluid.

Parameter	Bacterial meningitis	Viral (Aseptic) meningitis
Cell count	Elevated (1000/mm³), predominantly PMNs	Mild to moderate increase, lymphocytes
Protein	Increased	Mildly increased
Glucose	Decreased	Normal
CSF appearance	Turbid/purulent	Clear

Chronic Meningitis

Chronic meningitis is more commonly observed in immunocompromised individuals, although it can also occur in immunocompetent patients. Nausea, vomiting, headache, stiff neck, and fever, lethargy, confusion, and mental decline are some of the signs that individuals may experience as the disease develops slowly. Before seeking therapy, symptoms could last for a one month or longer. The CSF typically shows a reduction in glucose concentration, increased protein, and an abnormally high number of leukocytes are typically lymphocytic. Acute and chronic meningitis share a similar pathophysiology [20].

Aseptic Meningitis

Aseptic meningitis, which frequently has a viral origin, is characterized by pleocytosis, or an increase in lymphocytes and other mononuclear cells in the fluid; bacterial and fungal cultures do not exhibit any symptoms of the disease. On the other hand, bacterial meningitis is characterized by purulence and the polymorphonuclear (PMN) cell response in the fluid.

Aseptic meningitis is typically self-limiting and presents with symptoms including fever, headache, neck stiffness, nausea, and vomiting. Symptoms of meningeal irritation (meningism) include:

Pathognomonic sign: This sign of meningeal irritation is nuchal stiffness, sometimes referred to as "stiff neck," which occurs when the neck resists passive flexion.

Kernig's sign: When the hip is extended to 90 degrees, and the leg cannot be straightened due to severe hamstring stiffness.

Brudzinski's sign: The hips and knees flex spontaneously while the neck is passively flexed.

Infants with pyogenic meningitis may experience a delayed start, nonspecific symptoms, and no neck stiffness. Babies typically arrive with a swollen fontanelle, fever, and irritability. Over time, the illness may affect the brain parenchyma, resulting in meningoencephalitis, which can cause seizures, elevated intracranial pressure, stroke, and diminished awareness. Purpuric rashes are one particular symptom of meningococcal meningitis that can occasionally be observed [22].

Causative agent

Most people think of bacterial meningitis as a childhood illness. These disorders have a high incidence and fatality rate in the majority of babies and children. With high and widespread incidence between 2% and 30%, bacterial meningitis remains a nightmare despite advances in antibacterial drugs and critical care [23]. The patient's age and geographic location have a major impact on the etiology of this potentially fatal illness. In general, group B streptococci (GBS), *Escherichia coli, *and *Klebsiella pneumoniae* are commonly linked etiologic agents of bacterial meningitis in newborns [24].

Before the vaccination was introduced, 90% of cases of meningitis in children under five years of age were caused by the most frequent bacteria, Meningococcus, Pneumococcus, and Hib [25]. According to a Greek study on the prevalence of bacterial meningitis in children, *N. meningitidis* was the causal organism, and the majority of cases occurred in newborns and early children [26].

Before the pentavalent vaccine was introduced in India, it was reported that Hib was the primary cause of meningitis in children with high mortality [27]. It was discovered to be the most common cause of bacterial meningitis in infants younger than two in several Indian hospitals between 2008 and 2010, followed by *S. pneumoniae *and group B Streptococcus, respectively [28]. But between 2011 and 2015, *S. pneumoniae* was found to be the primary reason for meningitis caused by bacteria in children under five years of age [29]. Table 2 shows the common etiological agents of acute bacterial meningitis by age group.

**Table 2:** Common etiological agents of acute bacterial meningitis, by age group This table shows the distribution of common meningitis-causing pathogens by age [23-25,28,29]. Group B Streptococci (GBS), *Escherichia coli,* and *Klebsiella pneumoniae *are the most frequent causes of infections in newborns. *Neisseria meningitidis, Streptococcus pneumoniae, and Haemophilus influenzae type B *are the pathogens that affect newborns and children. Gram-negative bacteria, *Staphylococcus species*, and *Streptococcus pneumoniae* and* Neisseria meningitidis *are the most common bacteria in adults. Gram-negative bacteria and *Listeria monocytogenes *are more frequently linked to immunocompromised or elderly people.

Age group	Common pathogens
Neonates	Group B Streptococci (GBS), *Escherichia coli, Klebsiella pneumoniae*
Infants & children	Haemophilus influenzae type b, Neisseria meningitidis, Streptococcus pneumoniae
Adults	*Streptococcus pneumoniae, Neisseria meningitidis, Staphylococcus spp.,* Gram-negative bacilli
Elderly / Immunocompromised	*Listeria monocytogenes, *Gram-negative bacilli

Bacterial meningitis is an inflammation of the meninges caused by bacterial infection. *H. influenzae* (40%-60%) and *S. pneumoniae *(10%-20%) are among the most commonly reported causes of meningitis in children [25,26]. In adults, *S. pneumoniae* (30%-50%), *N. meningitidis *(10%-35%), *Staphylococcus *(5%-15%), other *Streptococcus *species, *H. influenzae* (1%-3%), Gram-negative bacilli (1%-10%), and *L. monocytogenes* are major pathogens associated with bacterial meningitis [1,2,26].

Clinical significance of meningitis

Early meningitis symptoms in young infants are frequently ill-defined and ambiguous. Generally speaking, the younger the baby, the less specific the symptoms are. Fever (with or without vomiting), behavioural changes (baby becomes drowsy or lethargic, irritable, and feeds poorly), a high-pitched cry, seizures, and a full or stiff anterior fontanelle are the key symptoms that strongly suggest an infant has ABM.

Infants under the age of two rarely exhibit specific symptoms of meningeal inflammation. Classic meningitis symptoms, such as fever, headache, vomiting, photophobia, stiff neck, and meningeal signs, are likely to be present in older children of all the meningeal symptoms, neck stiffness is the most significant and first to manifest. If the patient is checked sitting up with their knees out, it becomes more noticeable.

Other meningeal indications include the Brudzinski and Kernig signs. Reflex muscle spasm in response to pain when the spinal cord's contents are stretched is what causes the meningeal symptoms. Patients in a coma may not exhibit these symptoms. The second form of presentation is acute and fulminant, characterized by significant cerebral edema, elevated ICP, and quickly developing meningitis and sepsis symptoms. This is typically caused by kind of presentation is *N. meningitidis.* While they can also happen in meningitis caused by other organisms, petechial hemorrhage that quickly coalesce and produce purpura patches on the skin are believed to be a hallmark of this illness. Numerous series have described cases of lethal shock and profound hypotension [30,31].

About 30%-40% percent of ABM cases result in seizures. The incidence of seizures has been linked to elevated levels of tumour necrosis factor (TNF) [32]. Increased ICP, cerebritis, or hypotension are the common causes of altered mental status and decreased awareness. In cases of simple acute meningitis, papilledema is rare and indicates a more chronic condition, such as an intracranial abscess, subdural empyema, or dural venous sinus blockage. Vascular blockage, abscess formation, or cortical infarction can all cause focal neurologic symptoms. Overall, focal neurological symptoms are present in 14% of kids with meningitis caused by bacteria [33]. Table 3 shows the common clinical features of acute bacterial meningitis.

**Table 3:** Common clinical features of acute bacterial meningitis Clinical features of meningitis categorized by symptom type [30-33]. Fever, headaches, nausea, and impaired mental status are examples of general symptoms. Neck stiffness and positive Kernig's and Brudzinski's signs are examples of meningeal signs. Seizures, diminished consciousness, and focal neurological impairments are examples of neurological symptoms. Features that are typical of babies include poor eating, irritability, and a protruding fontanelle.

Category	Clinical features
General symptoms	Fever, headache, vomiting, altered mental status
Meningeal signs	Neck stiffness, Kernig’s sign, Brudzinski’s sign
Neurological signs	Seizures, decreased consciousness, focal deficits
Infant-specific signs	Bulging fontanelle, irritability, poor feeding

Pathogenesis of bacterial meningitis

High-grade bacteremia is thought to typically precede bacterial meningitis, and the germs enter the central nervous system from the bloodstream. Local infections or dural abnormalities are the other possible entry points.

Mechanism

Meningitis-causing bacteria first colonize the nasopharynx by adhering to the nasopharyngeal epithelial cell. Bacteria are then carried to the intravascular space via epithelial cells. Once in the circulation, they are shielded from neutrophil phagocytosis and traditional complement-mediated bactericidal action by a polysaccharide capsule. These bacteria can now infiltrate the intraventricular choroid plexus, infect the choroid plexus's epithelial cells, and eventually reach the CSF.

The hallmarks of bacterial meningitis include pleocytosis and enhanced BBB permeability, which are brought about by the discharge of toxic and pro-inflammatory chemicals by bacteria. Matrix metalloproteinases (MMPs), chemokines, nitric oxide, peroxynitrite, cytokines and reactive oxygen species, tumor necrosis factor-α (TNF-α), transforming growth factor-β1 (TGF-β1), arachidonic acid metabolites, platelet activating factor (PAF), proinflammatory neuropeptides, and caspases are mediators that increase BBB permeability and facilitate white blood cell (WBC) migration. Bacterial phagocytosis and poor opsonization are caused by the very modest amounts of complement protein, immunoglobulin, and WBC in CSF [34].

The inflammatory response caused by the invasive bacteria is the important event in the pathogenesis. Therefore, even after antibiotic therapy has sterilized the fluid, neurological damage may still worsen. When bactericidal antibiotics are used, bacteria will lyse and release components of their cell walls, such as lipopolysaccharide, teichoic acid, and peptidoglycans, which cause meningeal inflammation by producing inflammatory cytokines and chemokines. This leads to four distinct features: modified permeability of the BBB, leukocyte adhesion to cerebral capillary endothelial cells, modification of cerebral circulation, the cohort of nitrogen species (NS), reactive oxygen (RO), and excitatory amino acids.

Vasogenic edema, obstructive and communicative hydrocephalus, interstitial edema, cytotoxic edema, cell damage, and death are the ultimate outcomes of all the aforementioned pathways. Increased ICP and coma result from the combination of interstitial, vasogenic, and cytotoxic enema [35]. Table 4 shows the mechanism of pathogenesis in ABM.

**Table 4:** Mechanism of pathogenesis in acute bacterial meningitis Sequential stages in the bacterial meningitis pathogenesis [34-46]. Nasopharyngeal colonization is the first step in the process, which is then followed by bloodstream invasion and survival by eluding the host's immune system. Then, through endothelial interactions, bacteria get through the blood–brain barrier (BBB) and enter the cerebrospinal fluid (CSF), where low immune activity promotes fast multiplication. This causes a severe inflammatory reaction that releases mediators and cytokines, disrupting the blood-brain barrier, increasing permeability, and causing edema. Toxic and inflammatory pathways cause subsequent neuronal damage, which leads to clinical consequences such elevated intracranial pressure, cerebral edema, convulsions, coma, and neurological impairments.

Step	Mechanism	Key events
Colonization	Nasopharyngeal adherence	Bacteria attach to epithelial cells using surface adhesins
Bloodstream invasion	Entry into circulation	Bacteria evade immune response via polysaccharide capsule
Survival in blood	Resistance to host defenses	Avoid phagocytosis and complement-mediated killing
Blood-brain barrier (BBB) crossing	Endothelial interaction	Binding to receptors (e.g., PAF receptor) enables penetration
CSF invasion	Rapid multiplication	Low immune activity in CSF allows bacterial growth
Inflammatory response	Cytokine and mediator release	TNF-α, interleukins, nitric oxide, and chemokines released
BBB disruption	Increased permeability	Leads to leukocyte migration and edema formation
Neuronal injury	Toxic and inflammatory damage	Release of bacterial toxins, oxidative stress, apoptosis
Clinical complications	Raised ICP and brain damage	Cerebral edema, seizures, coma, neurological deficits

Bacterial Invasion

The choroid plexus may be the anatomical location of bacterial infection, according to Daum et al. (1978) [36]. In order to get across the BBB, meningeal infections possess efficient molecular tools. For example, Streptococcal proteins like CbpA (choline-binding protein) interact with phosphorylcholine glycoconjugate receptors with platelet activating factor (PAF) on eukaryotic cells to facilitate endocytosis and BBB crossing [37-40].

Inflammatory Response

In addition to regulating adhesion molecules like Intracellular Adhesion Molecules (ICAM-1), endothelial cell activation appears to be the first stage of bacterial invasion [41]. These adhesion molecules then start a number of leukocyte invasion processes. Granulocytes in the fluid are recognized as the diagnostic hallmark of meningitis. Matrix metalloproteinases (MMPs) and nitric oxide (NO), which are byproducts of active leukocytes, appear to develop concurrently with the inflammatory response and bacterial invasion [42,43]. This causes the BBB and blood cerebrospinal fluid barrier to malfunction. Autolysis brought on by bacterial reproduction exacerbates the inflammation.

Neuronal Injury

Meningitis causes neuronal damage due to numerous mechanisms, such as bacterial toxins, cytotoxic products of immune-competent cells, and other diseases that are connected to intracranial problems. Pneumolysin, a pore-forming cytolysin, and H2O2 are two significant toxins that induce the majority of neuronal damage in infections by *S. pneumoniae*, for example [44,45].

Brain injury and intracranial problems are caused by two main pathways, which involve bacterial toxins and cytotoxic products of the inflammatory response, such as apoptosis inducing factor (AIF), lipopolysaccharide (LPS), bacterial lipopeptide (LP), peptidoglycan (PG), and ICP [46].

## Review

Methods

A comprehensive literature search was performed using electronic databases, including PubMed, Google Scholar, and Scopus. The search strategy incorporated keywords such as “acute bacterial meningitis,” “cerebrospinal fluid,” “antibiogram,” and “phenotypic characterization,” combined using appropriate Boolean operators (AND, OR). Studies published in English and relevant to CSF analysis, bacterial pathogens, and antibiotic resistance patterns were included. Both observational studies and original research articles were considered. Review articles, case reports with insufficient data, and studies not related to bacterial meningitis were excluded.

**Figure 1:**
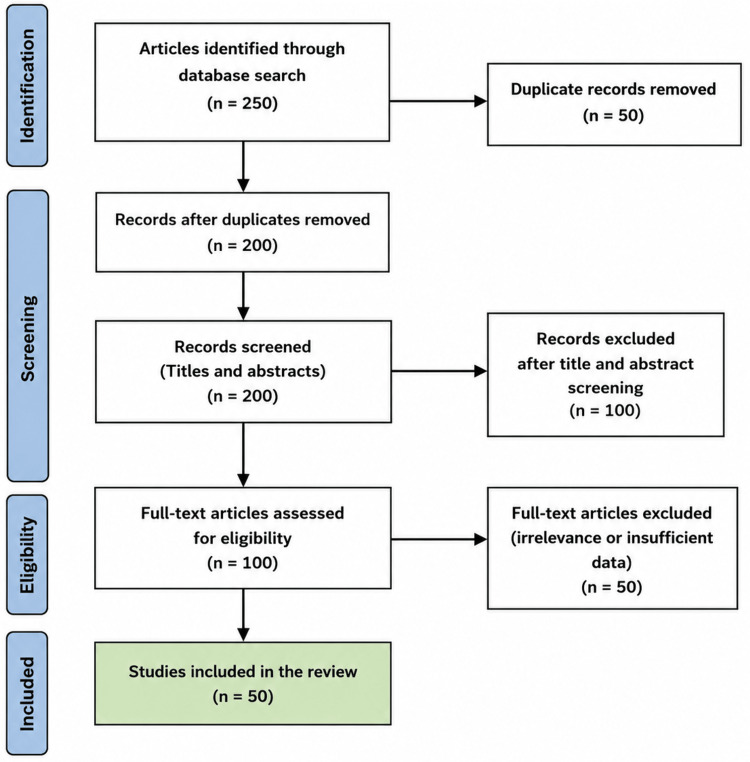
PRISMA flow chart of study selection n: Number, PRISMA: Preferred Reporting Items for Systematic Reviews and Meta-Analyses

Titles and abstracts were initially screened, followed by full-text evaluation for eligibility. Duplicate records were identified and removed during the screening process. Data extracted from the selected studies included study design, population characteristics, types of bacterial isolates, and antibiotic susceptibility patterns. A total of 250 articles were identified through this database search. After removal of duplicates, 200 articles remained for screening. Following title and abstract screening, 100 articles were excluded. The full texts of 100 articles were assessed for eligibility, of which 50 were excluded due to irrelevance or insufficient data. Finally, 50 studies were included in the review. This review was conducted as a narrative synthesis of the available literature; therefore, no quantitative meta-analysis was performed. The study selection process is illustrated in a Preferred Reporting Items for Systematic Reviews and Meta-Analyses (PRISMA) [47] flow diagram (Figure 1).

Results

Microbiological Diagnosis, Phenotypic Characterization, and Antibiotic Susceptibility Testing (AST)

Across the studies included in this review, CSF samples were processed using conventional microbiological methods for the diagnosis of acute bacterial meningitis. Initial evaluation commonly included Gram staining followed by culture on blood agar, chocolate agar, and MacConkey agar depending on the suspected organism [48,49].

Phenotypic identification was primarily based on colony morphology, staining characteristics, and biochemical tests. *S. pneumoniae* was identified by alpha-hemolytic colonies, optochin sensitivity, and bile solubility testing [5,49]. *N. meningitidis* was characterized as Gram-negative diplococci with oxidase positivity and growth on chocolate agar [6]. *H. influenzae* required X and V growth factors for isolation [3]. Staphylococcal species were differentiated using catalase and coagulase tests, while Gram-negative bacilli were identified using standard biochemical reactions [48,49].

AST was most commonly performed using the Kirby-Bauer disk diffusion method, although some studies also utilized minimum inhibitory concentration (MIC) techniques [5,49]. Interpretation of AST results was performed according to Clinical and Laboratory Standards Institute (CLSI) guidelines where reported [3].

Analysis of the reviewed studies demonstrated that *S. pneumoniae *remains the leading cause of ABM, followed by *N. meningitidis* [1,5,6,26,48,50]. Gram-negative organisms were predominantly reported in neonatal populations, whereas adult cases showed a higher prevalence of pneumococcal infections. Overall, the distribution of pathogens varied according to age group and geographic setting, although a consistent predominance of these organisms was observed across most studies [23-29].

Evaluation of the antibiogram findings demonstrated increasing resistance to commonly prescribed beta-lactams and macrolides [2,5,49]. In contrast, third-generation cephalosporins, vancomycin, and carbapenems retained comparatively better antimicrobial activity against most isolates, although multidrug resistance among Gram-negative bacilli was also reported [2,5,48,49].

Specifically, *S. pneumoniae* demonstrated resistance to penicillin and erythromycin while remaining largely susceptible to ceftriaxone and vancomycin [2,5,49]. *N. meningitidis* showed occasional reduced susceptibility to penicillin but retained sensitivity to third-generation cephalosporins [2,49]. Gram-negative bacilli exhibited resistance to third-generation cephalosporins, often due to extended-spectrum beta-lactamase (ESBL) production, but remained susceptible to carbapenems and aminoglycosides [5,48].

These findings emphasize the importance of continuous surveillance of antimicrobial susceptibility patterns and antibiogram-guided empirical therapy in ABM. Table 5 summarizes the general trends in antibiotic resistance among CSF isolates.

**Table 5:** Antibiotic susceptibility patterns of common bacterial pathogens in CSF isolates Antibiotic susceptibility patterns among cerebrospinal (CSF) isolates demonstrate increasing resistance to commonly used antibiotics. *Streptococcus pneumoniae* shows significant resistance to penicillin and macrolides, while remaining largely susceptible to third-generation cephalosporins and vancomycin. *Neisseria meningitidis *generally remains sensitive to ceftriaxone and cefotaxime, although reduced susceptibility to penicillin has been reported in some regions. *Haemophilus influenzae* frequently produces beta-lactamase, leading to resistance to ampicillin, but retains susceptibility to cephalosporins and beta-lactamase inhibitor combinations. Gram-negative bacilli, including *Escherichia coli *and *Klebsiella species*, often exhibit multidrug resistance, particularly due to extended-spectrum beta-lactamase (ESBL) production, but are typically susceptible to carbapenems and aminoglycosides.

Organism	Resistant antibiotics	Susceptible antibiotics
Streptococcus pneumoniae	Penicillin, erythromycin, some cephalosporins	Ceftriaxone, vancomycin, linezolid
Neisseria meningitidis	Occasional reduced susceptibility to penicillin	Ceftriaxone, cefotaxime, rifampicin
Haemophilus influenzae	Ampicillin (beta-lactamase producers)	Ceftriaxone, amoxicillin-clavulanate
Gram-negative bacilli (*Escherichia coli, Klebsiella spp.*)	Ampicillin, third-generation cephalosporins (ESBL producers)	Carbapenems (meropenem, imipenem), amikacin

These findings demonstrate a shift toward increasing antimicrobial resistance, emphasizing the need for continuous surveillance and updated empirical treatment strategies.

Discussion

ABM remains a major cause of morbidity and mortality despite advances in antimicrobial therapy and vaccination programs [1,2]. This review demonstrated that *S. pneumoniae* remains the most common causative organism of ABM, followed by *N. meningitidis*, which is consistent with previous epidemiological studies [1,5,6,26]. Neonatal meningitis was more commonly associated with Gram-negative bacilli such as *E. coli *and *K. pneumoniae,* whereas adult cases predominantly involved pneumococcal infections [23,24].

An important finding of this review is the increasing antimicrobial resistance among CSF isolates. *S. pneumoniae* showed considerable resistance to penicillin and macrolides, while most isolates remained susceptible to ceftriaxone and vancomycin [2,5,49]. Similarly, Gram-negative bacilli demonstrated resistance to third-generation cephalosporins due to ESBL production, although carbapenems retained good activity [5,48]. These findings highlight the importance of continuous surveillance and antibiogram-guided therapy in the management of ABM.

Conventional microbiological methods, including Gram staining, culture, and biochemical testing, continue to play a major role in the diagnosis and phenotypic characterization of bacterial pathogens, especially in resource-limited settings [48,49]. Characteristic CSF findings such as neutrophilic pleocytosis, elevated protein levels, and reduced glucose concentration remain essential for differentiating bacterial meningitis from viral meningitis [20-22].

The reviewed studies also emphasized the role of bacterial invasion and inflammatory responses in the pathogenesis of meningitis. Cytokine release, BBB disruption, and neuronal injury contribute significantly to cerebral edema, increased ICP, and neurological complications [34,41-44]. Even after bacterial clearance, inflammation may persist and worsen neurological damage [35].

Vaccination programs against Hib and *S. pneumoniae* have significantly reduced the incidence of bacterial meningitis in children [25,27,30]. However, the emergence of resistant strains and multidrug-resistant organisms continues to pose therapeutic challenges. Therefore, regular monitoring of resistance trends, rational antibiotic use, and strengthening vaccination coverage remain essential to reduce the burden of ABM.

Overall, this review highlights the continuing clinical significance of ABM and emphasizes the need for rapid diagnosis, accurate pathogen identification, and appropriate antimicrobial therapy to improve patient outcomes.

## Conclusions

Globally, ABM is still a major neurological illness that can be fatal and has a high morbidity and fatality rate. Improving patient outcomes is largely dependent on early diagnosis through CSF examination and timely beginning of suitable antibiotic medication. For precise pathogen identification and to direct both empirical and targeted treatment approaches, phenotypic characterisation and antibiotic susceptibility testing of CSF isolates are crucial. A significant obstacle to the efficient management of ABM is the growing incidence of antibiotic resistance among prevalent causing organisms. This emphasizes how crucial it is to monitor resistance patterns and use antibiogram-based treatment in clinical settings. The prevalence of some infections has been considerably decreased by preventive measures, such as mass immunization campaigns and public health education, although ongoing surveillance is still required.

Additionally, early detection and improved clinical decision-making can be facilitated by upgrading laboratory testing capabilities and implementing quick diagnostic techniques. The creation of innovative treatment methods and tactics to counteract antibiotic resistance should be the main emphasis of future research. To lessen the worldwide burden of ABM, a multidisciplinary strategy combining physicians, microbiologists, and public health officials is crucial.
